# Vivax malaria in pregnancy and lactation: a long way to health equity

**DOI:** 10.1186/s12936-020-3123-1

**Published:** 2020-01-22

**Authors:** Tobias Brummaier, Mary Ellen Gilder, Gornpan Gornsawun, Cindy S. Chu, Germana Bancone, Mupawjay Pimanpanarak, Kesinee Chotivanich, François Nosten, Rose McGready

**Affiliations:** 1 Shoklo Malaria Research Unit, Mahidol–Oxford Tropical Medicine Research Unit, Faculty of Tropical Medicine, Mahidol University, P.O. Box 46, 68/31 Bann Tung Road, Mae Sot, 63110 Thailand; 2 Swiss Tropical and Public Health Institute, Basel, Switzerland; 3 University of Basel, Basel, Switzerland; 4 Centre for Tropical Medicine and Global Health, Nuffield Department of Medicine, University of Oxford, Old Road Campus, Oxford, UK; 5 Mahidol–Oxford Tropical Medicine Research Unit, Faculty of Tropical Medicine, Mahidol University, Bangkok, Thailand

**Keywords:** *Plasmodium vivax*, Equity, Primaquine, Radical cure, G6PD deficiency

## Abstract

**Background:**

The Sustainable Development Goals (SDG) call for increased gender equity and reduction in malaria-related mortality and morbidity. *Plasmodium vivax* infections in pregnancy are associated with maternal anaemia and increased adverse perinatal outcomes. Providing radical cure for women with 8-aminoquinolines (e.g., primaquine) is hindered by gender-specific complexities.

**Case presentation:**

A symptomatic episode of vivax malaria at 18 weeks of gestation in a primigravid woman was associated with maternal anaemia, a recurrent asymptomatic *P. vivax* episode, severe intra-uterine growth restriction with no other identifiable cause and induction to reduce the risk of stillbirth. At 5 months postpartum a qualitative glucose-6-phosphate dehydrogenase (G6PD) point-of-care test was normal and radical cure with primaquine was prescribed to the mother. A 33% fractional decrease in haematocrit on day 7 of primaquine led to further testing which showed intermediate phenotypic G6PD activity; the G6PD genotype could not be identified. Her infant daughter was well throughout maternal treatment and found to be heterozygous for Mahidol variant.

**Conclusion:**

Adverse effects of vivax malaria in pregnancy, ineligibility of radical cure for pregnant and postpartum women, and difficulties in diagnosing intermediate levels of G6PD activity multiplied morbidity in this woman. Steps towards meeting the SDG include prevention of malaria in pregnancy, reducing unnecessary exclusion of women from radical cure, and accessible quantitative G6PD screening in *P. vivax*-endemic settings.

## Background

The Sustainable Development Goals (SDG) highlight freedom from the adverse effects of malaria (SDG 3.3) and gender equity (SDG 4) as global priorities for this decade. The intersection between health and gender equity has been recognized, and gender-specific inequities in the treatment and prevention of malaria have been a focus of recent quantitative and qualitative research [1]. Gender equity in health care requires not only equal access to health services, but also *unequal access* in response to *unequal burden*. In other words, one of the principles of gender equity requires that services for women not simply mirror services for men but differ from services for men in situations where women’s needs or disease processes differ [2].

The World Health Organization (WHO) estimated the global incidence of *Plasmodium vivax* to be 7.5 million cases in 2017, and it is the most prevalent malaria species in Southeast Asia [3]. Women and their fetuses uniquely suffer from the complications of malaria in pregnancy [4, 5], including those associated with *P. vivax* infection [6].

*Plasmodium vivax* and *Plasmodium ovale* are the only two malaria species capable of relapses, due to the presence and subsequent activation of dormant liver stages known as hypnozoites. Treatment of the blood stages of acute vivax malaria relies on the schizontocidal agent chloroquine (CHQ) in most parts of the world, and is safe in pregnancy [7]. Relapse prevention is achieved with the 8-aminoquinolines primaquine (PMQ) or tafenoquine (TQ), both active against *P. vivax* hypnozoites [7, 8] and contra-indicated in pregnancy as the glucose-6-phosphate dehydrogenase (G6PD) status of the fetus cannot be determined antenatally in most malaria endemic settings.

The use of PMQ for radical cure is complicated by a few factors, including adherence to a 14-day course, commonly observed side effects such as abdominal pain and, specifically in G6PD-deficient individuals, the risk of drug-induced haemolysis [9–12]. TQ provides radical cure in a single dose, improving adherence, but has not yet been implemented widely since receiving approval from the US Food and Drug Administration (FDA) in 2018 [13]. A major barrier to TQ roll-out is the poor sensitivity of common qualitative point-of-care tests to detect intermediate levels of G6PD deficiency in heterozygote females who are at risk for haemolysis.

G6PD deficiency is caused by mutations of the G6PD gene, which is located on the X-chromosome; genotypes and phenotypic expression are different in males and females. Males are either hemizygous wild types (with a normal phenotype) or hemizygous mutated (with a deficient phenotype), and females can be homozygous mutated (with a deficient phenotype), homozygous wild type (with a normal phenotype) or heterozygous. The G6PD enzymatic activity of heterozygous females includes a spectrum of activity from partial deficiency to normal [14]. With qualitative G6PD rapid tests, patients can only be classified as deficient or normal according to the test threshold [15, 16]. As a result, heterozygous females with intermediate enzymatic activity are usually diagnosed as G6PD normal even though they are susceptible to PMQ- and TQ-induced haemolysis [17]. If qualitative G6PD tests are done in the presence of anaemia or a haemolytic episode, false normal results are more likely, especially for heterozygote females [18]. Some point-of-care quantitative G6PD tests have now been validated in laboratory settings [19, 20] but field validation for use in malaria case management has not yet been completed.

As there are no safe options for radical cure during pregnancy, the WHO conditionally recommends radical cure postpartum, after the infant has reached 6 months of age [7, 21]. On the Thailand-Myanmar border the strongest risk factor for *P. vivax* in the first 12 weeks post-partum was a history of *P. vivax* infection during the previous 9 months [22]. Exclusion of pregnant and lactating females from radical cure potentially affects about 13% of females in malaria-endemic areas [6]. Providing PMQ postpartum for radical cure of *P. vivax* is essential in Southeast Asia because the majority of *P. vivax* infections are attributed to relapse [23, 24]. In the absence of timely radical cure, relapses may cause illnesses and anaemia in the postpartum period, and deleterious effects in a subsequent pregnancy, increasing the risk of maternal morbidity (mostly due to anaemia), fetal loss (due to miscarriage and stillbirth) and neonatal mortality (due to pre-term birth, intra-uterine growth restriction (IUGR) and low birth weight) [4, 5, 25].

The aim of reporting this case is to describe the multiple gender-specific morbidities suffered by a woman infected with *P. vivax* during pregnancy and to discuss potential research and programmatic priorities that could improve gender equity in *P. vivax* prevention and treatment.

## The case

### History and examination

An 18-year-old, primigravid, pregnant Burman woman with an unremarkable medical history, presented to the antenatal care (ANC) clinic of Shoklo Malaria Research Unit (SMRU) on the Thailand-Myanmar border. She reported a 7-day history of fever, chills, rigour, headache, dizziness, occasional palpitations, general weakness as well as muscle and joint pain. She used an impregnated bed net nightly and denied having any previous malaria infection. Obstetric ultrasound examination confirmed a viable fetus with an estimated gestational age (EGA) of 18 weeks and 4 days. The peripheral blood smear was positive for *P. vivax* (Table 1).

**Table 1:** Vital signs and blood profiles at the first and second *Plasmodium vivax* episode

	1st episode	2nd episode
Schizontocidal treatment	COA+	CHQ
EGA (weeks + days)	18 + 4	35 + 4
Maternal vital signs		
Heart rate (beats per minute)	70	92
Blood pressure (mmHg)	110/60	120/80
Respiratory rate (per minute)	24	23
Body temperature (degree celsius)	38.8	36.1
Weight (kg)	57	66
Fetal heart rate (beats per minute)	148	152
Fundal height (cm)	15	29
PVT (per µL)	340	478
PVG (per µL)	136	62
Parasite clearance (days)	1	2
RBC (× 10^6^/μL)	3.63 (2.81–4.49)	4.0 (2.71–4.73)
HCT (%)	31.2* (30–39)	35.1 (28.0–40.0)
HB (g/dL)	10.0 (9.7–14.8)	11.9 (9.5–15.0)
Reticulocytes (%)	5.4 (0.8–2.0)	NA
WBC (× 10^3^/μL)	8.5 (5.6–14.8)	10.4 (5.9–16.9)
Platelets (× 10^3^/μL)	245 (155–409)	217 (146–429)

Reference ranges are shown in brackets and were adjusted to the corresponding trimester [38]

CHQ, Chloroquine; COA+, augmented regimen of artemether–lumefantrine (5 tablets twice daily for 4 days); EGA, estimated gestational age; HB, haemoglobin; HCT, haematocrit; NA, not available; PVG, *Plasmodium vivax* gametocytes; PVT, *Plasmodium vivax* trophozoites; RBC, red blood cell count; WBC, white blood cell count

* HCT declined to 27% on day 5

### Laboratory findings

Haematologic laboratory parameters on admission showed a haematocrit (HCT) of 31.2% and haemoglobin of 10 g/dL. The reticulocyte count was elevated at 54 per 1000 red blood cells (normal range 8–20) indicating increased erythropoiesis. Haemoglobin typing, G6PD fluorescent spot test (FST), oral glucose tolerance test were normal and VRDL, HIV and urine culture were negative.

### Treatment

The patient consented to participate in a randomized, controlled, treatment trial (ClinicalTrials.gov Identifier NCT01054248) and received an augmented regimen of artemether–lumefantrine (COA+) with 5 tablets twice daily for 4 days (each dose contained 100 mg/600 mg artemether/lumefantrine). As per WHO recommendations for pregnant women, PMQ was not given. Chloroquine chemoprophylaxis was not given following treatment with COA+ as this was not in the trial protocol.

Parasitaemia cleared after 1 day of treatment and no fever was recorded on 6-hourly temperature measurements. The patient was discharged on day 5. Since the HCT value on the day of discharge was only 27%, the patient was prescribed anaemia treatment (ferrous sulfate 400 mg twice daily and folic acid 5 mg once daily). The patient followed ANC once weekly for 63 days as per study protocol. Malarial screening was repeatedly negative and the HCT rose to 33% after 2 weeks of anaemia treatment.

Nearly 5 months later, at an estimated gestational age of 35 + 4 weeks, routine malaria screening by microscopy during the ANC visit detected *P. vivax*. She had no symptoms and was treated with CHQ (10 mg/kg per day for 2 days and 5 mg/kg per day for 1 day) following the standard treatment recommended by the WHO [7]. Parasites cleared after 2 days of CHQ and the HCT (35%) was normal. As with the first *P. vivax* episode, no prophylaxis was given.

### Delivery

Routine symphysis fundal height measurement indicated poor fetal growth, which was confirmed by fetal anthropometry using ultrasound. After induction of labour for severe IUGR, a normal female infant was birthed in an SMRU clinic with an estimated gestational age of 38 + 6 weeks. The Apgar scores were 9 and 10 at 1 and 5 min, respectively. On examination, small for gestational age (< 1st centile by international standards) was confirmed with a body weight of 1980 g, a head circumference of 30 cm (< 3rd centile) and a body length of 45 cm (< 10th centile) [26]. Maternal, neonatal, umbilical cord, and placental blood smears were negative for malaria parasites. No risk factors other than the *P. vivax* episodes during pregnancy were identified for the severe IUGR.

### Postpartum radical cure

After delivery the mother complied with study follow-up visits and no more complications arose. At 5 months postpartum the young mother was planning to move to a remote region with limited access to adequate health care. Radical curative treatment with PMQ for *P. vivax* was recommended before she moved, given the risk of anaemia and poor outcome of a subsequent pregnancy, despite being 1 month short of the WHO recommendation at that time (2016) for lactating mothers of 6 months [7].

Before administration of PMQ, the mother and infant were tested for G6PD deficiency using a FST; both were confirmed phenotypically normal. At baseline, the mother’s HCT was 41% [reticulocytes 3/1000 red blood cells (RBCs)] and the infant’s HCT was 36%; both blood slides were negative for *P. vivax* and the physical examination was unremarkable.

Daily weight-based treatment with PMQ (0.5 mg/kg/day) for 14 days total was prescribed; the 4 tablets daily translated into an actual dose of 0.52 mg/kg/day (weight 58 kg). The first dose was supervised in the clinic. At follow-up on the 7th day none of the commonly observed PMQ side effects, such as abdominal pain, nausea or vomiting was reported [9] but the patient complained of dizziness. The HCT was 27.4% (reference range for non-pregnant adult female 35.4–44.4%), an absolute reduction of 13.6% (from 41 to 27.4%), and equivalent to fractional reduction of 33%. Acute anaemia was confirmed with additional haematologic parameters and the patient was admitted for observation (Table 2).

**Table 2:** Blood results before, during, and after primaquine radical cure at 5 months postpartum

	Day 1 (before PMQ)	Day 4	Day 7	Day 10–12	11 weeks	7 months
HCT (35.4–44.4%)	41%	–	27.4%	30%	37%	36.1%
Reticulocytes (per 1000 RBC)	3	–	40	–	–	–
RBC (4.00–5.20 × 106/μL)	–	–	3.14	–	–	–
Hb (12.0–15.8 g/dL)	–	–	8.9	–	–	11.9
G6PD FST	Normal	–	–	–	–	–
G6PD activity* (IU/gHb) [% population median]	–	–	7.7 [103%]	–	–	4.62 [62%]
Infant HCT	36%	38%	33%	33%	–	36%
Infant G6PD FST	Normal		–	–	–	–

* Activity measured by spectrophotometry

Biochemistry showed normal kidney function and, apart from a mildly increased direct bilirubin (0.84 mg/dL [normal range: 0.3–1.3]) and alkaline phosphatase (132 U/L [33–96]), normal liver function tests. At 236 U/L (115–221) lactate dehydrogenase was slightly elevated. The direct Coombs test was negative and there was no haemoglobinuria. Otherwise, history and physical examination were unremarkable.

Given the clinical picture, G6PD deficiency was suspected despite the normal G6PD FST result as the FST has poor sensitivity to detect intermediate G6PD activity levels. The G6PD activity was then quantified by spectrophotometry and was found to be normal at 7.7 IU/gHb (population median: 7.51 IU/gHb) [27], suggesting a false normal result in a haemolytic state (Hb 8.9 mg/dL).

The patient’s body weight was rechecked and found to be 56.5 kg. With no signs of severe haemolysis and no evidence of G6PD deficiency, the PMQ course was continued under supervision at a corrected dose of 3.5 tablets daily (actual dose was 0.46 mg/kg/day for the remaining 7 days of treatment). The treatment was well tolerated from this point onwards and the woman’s clinical condition improved. To treat the anaemia, the patient was provided with a 28-day course of ferrous sulfate, folic acid and vitamin B12. Since the mother was still breastfeeding, the infant’s HCT was also followed and remained relatively stable (day 0, 4, 7 and 10 the HCT was 36, 38, 33 and 33%, respectively, fractional reduction of 8%). On day 12 of PMQ, the maternal HCT had increased to 30%, symptoms had resolved, and the patient requested to be discharged home. 11 weeks later, the HCT was 37%, and the patient was well. The child’s vaccination schedule was completed, and the 6-month motor milestones were normal.

### Additional laboratory findings following radical cure

Approximately 1 year after delivery, the mother returned with the infant for follow-up as part of the trial described earlier. Since the previous quantification of the G6PD activity during the haemolytic episode was considered unreliable (due to increased reticulocyte count and low Hb level), the staff offered the woman to repeat quantitative G6PD testing while in steady state (HCT 36.1%). This time the G6PD activity was found to be 4.62 IU/gHb, which corresponds to 62% of the population mean and is highly suggestive of G6PD heterozygosity [28]. Following this new finding, the patient and infant were screened for the most common G6PD gene mutations seen in the area (Mahidol, Chinese-4, Viangchan, Mediterranean variants) [29]. The coding regions from exon 2 to exon 13 of the G6PD gene of the mother were then sequenced according to protocol from Kim et al. [30] but no mutation was found. Mutations in non-coding regions have been linked to deficient G6PD activity [31]; however, in this case no further investigations were pursued. Interestingly the female infant was found to be heterozygous for the Mahidol variant. CYP2D6 genotyping was not performed but could have helped to clarify the haemolytic pattern.

## Conclusion

Recognizing gender-specific aspects of disease acquisition, detection, treatment options, and response to treatment can help ensure that health policies are effective and equitable. As the historical legacy of gender-based inequality is deeply embedded in medical research and the health sector, action to prevent undue gender-specific disparities in health outcomes is an ethical imperative and mandated in the SDG [32].

**Fig. 1:**
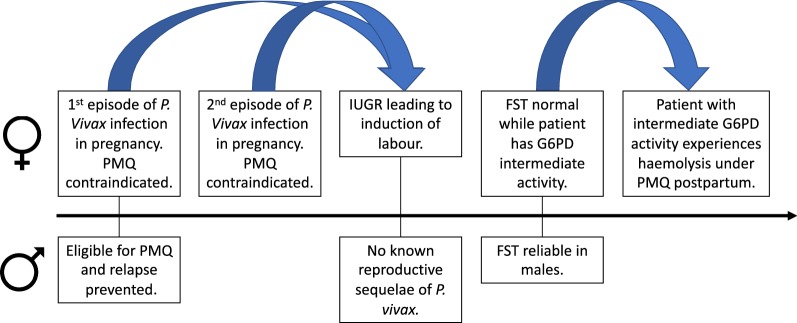
Comparison of timeline of *Plasmodium vivax* infection in this pregnant woman to a hypothetical male patient with *Plasmodium vivax*

In this case report of a female experiencing *P. vivax* in pregnancy, multiple layers of a gender-specific morbidity occur (Fig. 1). The reproductive impact of malaria is limited to females, and they suffer a double burden of more severe personal illness and poor outcomes for their offspring. The negative effect of *P. vivax* infection, especially recurrent infections, on birth weight has been demonstrated, and this increases both short- and long-term morbidity and mortality for affected infants [5].

The second layer of a gender-specific morbidity results from the ineligibility of many females for radical cure due to their reproductive stage. It is estimated that about 13% of females with *P. vivax* are temporarily ineligible for radical cure due to pregnancy or lactation [6], and the proportion who eventually receive 8-aminoquinolones postpartum as recommended by the WHO is unknown. Relapse is the main source of *P. vivax* infection in areas of low endemicity and contributes to chronic anaemia. A recent metanalysis confirms that, despite short term risk of haemolysis, radical cure with primaquine improves hematologic outcomes in the long term [33]. In most *P. vivax*-endemic regions, anaemia is a major public health problem, which contributes to maternal mortality [34]. The only strategies currently available to prevent harm from recurrent vivax malaria in pregnancy are weekly chemoprophylaxis with CHQ in pregnant women following their first episode of malaria, as recommended by the WHO, and early detection and treatment. Compelling theoretical concerns of iatrogenic fetal anaemia or hydrops prevent the use of 8-amnioquinolones in pregnancy, but there are no actual reports of adverse pregnancy outcomes after inadvertent administration. A recent publication included a report of 52 women in Brazil treated with primaquine during pregnancy (some repeatedly) without adverse birth outcomes [35]. However, G6PD testing was not done on the infants, and it is not unlikely that they were all G6PD normal. As other haemolytic drugs (eg. nitrofurantoin, dapsone) are used in pregnancy when indicated, it is conceivable that 8-aminoquinolones could be used as well. However, establishing the safety of current or new hypnozoiticidal drugs would require a cautious and systematic approach, starting with in silica or ex-vivo models of trans-placental transfer. Establishing safety during breastfeeding is considerably more straightforward. Pharmacokinetic data suggest that the 6-month postpartum delay for primaquine is unnecessarily long as PMQ is excreted into mature breast milk in negligible amounts [36]. Further information is needed on safety of PMQ in the neonatal period, and there is currently no data on TQ and lactation. Information on 8-aminoquinolones in the neonatal period are urgently needed as the opportune moment for radical cure is post-partum before the woman leaves the birth facility. Our current institute policy is weekly chemoprophylaxis with CHQ until delivery after treatment of blood stages in women who experience a *P. vivax* infection, followed by radical cure with PMQ after 1-month post-partum. Prior to discharge from the perinatal care unit, the patients are reminded of the significance of radical cure, and given a follow-up appointment.

The final layer of gender specific morbidity relates to tests for G6PD deficiency. When females are not pregnant or breastfeeding and are eligible for 8-aminoquinolones, they are at higher risk for misdiagnosis of G6PD deficiency by current qualitative testing [16]. This case involved a woman with only moderately decreased G6PD activity at 62% of the population mean, resulting in moderate but symptomatic haemolysis. It is expected that a fluorescent spot test (or an equivalent RDT) would classify heterozygous females with a G6PD activity as low as 30–40% as G6PD normal. In fact, Bancone et al. found that 60% of G6PD heterozygous females were misdiagnosed by qualitative screening [28]. Due to the loss of G6PD-deficient cells to haemolysis during illness, heterozygote females with even less than 30–40% activity could be misdiagnosed as normal if the test is done during an acute *P. vivax* episode. Such a misdiagnosis could result in fatal haemolysis if 8-aminoquinolines are inappropriately prescribed to females with intermediate G6PD activity, which is an important consideration as the new single radical curative dose TQ has a long terminal half-life. Field-validated point-of-care quantitative G6PD tests are an urgent priority to meet the ambitious WHO target of reducing malaria by 90% by the year 2030 [37]. This is especially true in areas with high prevalence of G6PD deficiency in order to avoid iatrogenic morbidity and mortality for females. Testing should be offered when individuals are healthy, such as at a preconception visit at the community level or during a routine antenatal care visit.

In this case the mother received PMQ while she continued to breastfeed a female infant with a G6PD heterozygous genotype (Mahidol variant) without adverse effects on the infant. Given the pharmacokinetics of primaquine during lactation and the dose-dependent nature of primaquine-induced haemolysis, it is very unlikely that the clinically insignificant 8% fractional reduction in HCT seen in this infant is due to drug exposure via breastmilk. However, the fact that she was classified as G6PD normal by FST further highlights the need for improved G6PD testing modalities.

Improving care and prevention of malaria in pregnancy, reducing unnecessary exclusion of women from radical cure, and providing quantitative G6PD screening that is as accurate for females as it is for males are achievable steps towards the SDG for a more equitable and malaria-free world.

## Data Availability

Not applicable.

## Abbreviations

ALT: alanine aminotransferase
ANC: antenatal care
AST: aspartate aminotransferase
CHQ: chloroquine
COA+: augmented regimen of artemether–lumefantrine
EGA: estimated gestational age
FDA: food and drug administration
FST: fluorescent spot test
G6PD: glucose-6-phosphate dehydrogenase
HB: haemoglobin
HCT: haematocrit
IUGR: intra-uterine growth restriction
NA: not available
OD: once a day
PMQ: primaquine
PVG: *Plasmodium vivax* gametocytes
PVT: *Plasmodium vivax* trophozoites
RBC: red blood cell count
RDT: rapid diagnostic test
SDG: Sustainable Development Goals
SMRU: Shoklo Malaria Research Unit
TQ: Tafenoquine
WBC: white blood cell count
WHO: World Health Organization
